# Stressed to Death: The Role of Transcription Factors in Plant Programmed Cell Death Induced by Abiotic and Biotic Stimuli

**DOI:** 10.3389/fpls.2020.01235

**Published:** 2020-08-12

**Authors:** Rory Burke, Johanna Schwarze, Orla L. Sherwood, Yasmine Jnaid, Paul F. McCabe, Joanna Kacprzyk

**Affiliations:** School of Biology and Environmental Science, University College Dublin, Dublin, Ireland

**Keywords:** programmed cell death, abiotic stress, biotic stress, transcription factors, plants

## Abstract

Programmed cell death (PCD) is a genetically controlled pathway that plants can use to selectively eliminate redundant or damaged cells. In addition to its fundamental role in plant development, PCD can often be activated as an essential defense response when dealing with biotic and abiotic stresses. For example, localized, tightly controlled PCD can promote plant survival by restricting pathogen growth, driving the development of morphological traits for stress tolerance such as aerenchyma, or triggering systemic pro-survival responses. Relatively little is known about the molecular control of this essential process in plants, especially in comparison to well-described cell death models in animals. However, the networks orchestrating transcriptional regulation of plant PCD are emerging. Transcription factors (TFs) regulate the clusters of stimuli inducible genes and play a fundamental role in plant responses, such as PCD, to abiotic and biotic stresses. Here, we discuss the roles of different classes of transcription factors, including members of NAC, ERF and WRKY families, in cell fate regulation in response to environmental stresses. The role of TFs in stress-induced mitochondrial retrograde signaling is also reviewed in the context of life-and-death decisions of the plant cell and future research directions for further elucidation of TF-mediated control of stress-induced PCD events are proposed. An increased understanding of these complex signaling networks will inform and facilitate future breeding strategies to increase crop tolerance to disease and/or abiotic stresses.

## Introduction

**Figure 1:**
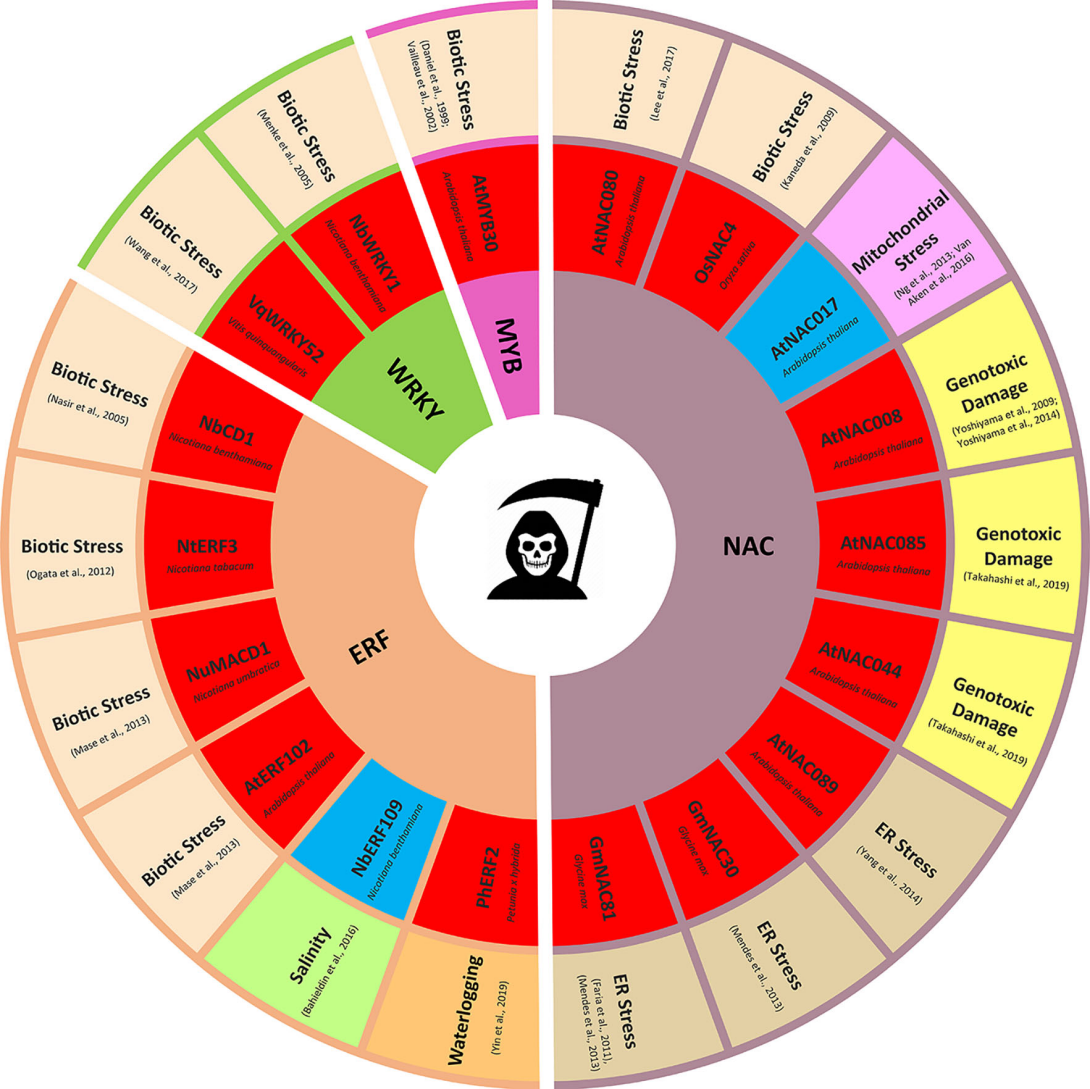
Transcription factors regulating stress induced programmed cell death (PCD). Only transcription factors (TFs) with experimentally validated role in PCD regulation are presented. TFs promoting PCD are highlighted in red, TFs suppressing PCD are highlighted in blue.

Programmed cell death (PCD) is a genetically controlled pathway of organized cell destruction (Danon et al., 2000). PCD is not only an essential element of plant development (Daneva et al., 2016), but also a part of the arsenal of defense responses against biotic and abiotic environmental stresses (Locato and De Gara, 2018). The classic example is the hypersensitive response (HR), a rapid cell death at the initial infection site activated to restrict the growth of biotrophic pathogens (Heath, 2000). Localized PCD events can also improve the plant’s ability to withstand abiotic stresses, for example, selective cell death triggered in the root stem cell niche was recently identified as an integral part of the cold acclimation process (Hong et al., 2017). Likewise, PCD plays a central role in plant adaptation to hypoxic conditions by mediating the formation of lysigenous aerenchyma, a porous tissue comprising internal spaces and channels to transport gases between plant shoots and roots (Evans, 2004). While aerenchyma formation is the key adaptive trait for waterlogging tolerance (Mustroph, 2018), it can also be induced under aerobic conditions by other abiotic stresses. Its formation converts living cortical tissue to air volume, thereby improving plant carbon economy, and reducing the respiratory and nutrient cost of soil exploration. Aerenchyma formation has also been reported to enhance nutrient stress adaptation (Fan et al., 2003; Saengwilai et al., 2014), as well as improve drought (Zhu et al., 2010) and salt (Saqib et al., 2005; Akcin et al., 2015) tolerance in different plant species. PCD can be also considered a protective mechanism when triggered by the excess excitation energy stress, leading to signal transduction to systemic cells and their acclimation to high light (Wituszyńska and Karpiński, 2013). However, PCD is not always beneficial to the plant: its activation can be an infection strategy for necrotrophic pathogens (Coll et al., 2011) and extensive PCD caused by severe abiotic stress may result in crop yield losses. Climate change is associated with increasing frequency of extreme weather events such as heavy rainfall, droughts, and heatwaves (USGCRP et al., 2017) that exacerbate abiotic stresses and plant diseases, challenging the global crop productivity. Therefore, there is a growing pressure to elucidate the complex regulatory networks behind plant pro-survival strategies, including those involving the tightly controlled activation of PCD in specific cells in response to environmental stimuli. Our understanding of plant PCD is still lagging behind that of animal cell death programs. Although recent progress in the field has identified a plethora of new PCD regulators, the complex molecular networks responsible for coordinating plant PCD are only just beginning to emerge (Daneva et al., 2016). In animals, the *bona fide* core PCD machinery is mainly regulated post-translationally (Fuchs and Steller, 2011), however, there are exceptions: *egl-1*, the key activator of the execution phase of apoptotic cell death in *Caenorhabditis elegans* (Horvitz, 2003) is expressed at a detectable level predominantly in cells programmed to die (Conradt et al., 2016). Additionally, the cell death pathway can be promoted and repressed by transcriptional regulators (Zhai et al., 2012; Aubrey et al., 2018). At least some level of transcriptional control of the cell death process is also likely in plants, where blocking transcription using *de novo* RNA synthesis inhibitor actinomycin D can both alleviate (Masuda et al., 2003; Vacca et al., 2004) and induce PCD (Ning et al., 2001). Transcription factors (TFs) are central players in eukaryotic gene regulation, binding to DNA in a sequence specific manner and promoting or inhibiting the activity of a transcription initiation complex (Voss and Hager, 2014). TFs may therefore act as molecular switches to regulate clusters of stimuli responsive genes (Pradhan et al., 2019). The involvement of major plant TF classes in a range of developmentally controlled PCD events was recently comprehensively discussed (Cubría-Radío and Nowack, 2019). Here, our aim is to discuss the role of TFs in PCD induced by various environmental stimuli, both abiotic and biotic in nature (**Figure 1**).

## NAC Transcription Factors

NAC TFs comprise one of the largest and most studied TFs families in plants. They contain a conserved DNA binding N-terminus and a more variable, transcription regulating, C-terminus (Ooka et al., 2003; Olsen et al., 2005). Several NACs have been linked to regulation of PCD triggered by abiotic and biotic stresses. NAC TFs have been implicated in regulation of the HR (Yuan et al., 2019). For example, OsNAC4 has been shown to positively regulate the HR by modulating the expression of almost 150 genes in rice (Kaneda et al., 2009). The OsNAC4 regulome included OsHSP90 and IREN, that act in parallel to induce HR PCD. Expression of *OsHSP90* is associated with the loss of plasma membrane integrity but not DNA fragmentation, while IREN, an endonuclease, is responsible for DNA degradation but alone does not affect plasma membrane integrity or induce cell death (Kaneda et al., 2009). The *Arabidopsis* NAC4 homologue, ANAC080 promotes cell death in response to bacterial infection by suppressing the transcription of three target genes; *LURP1*, *WRKY40*, and *WRKY54*, which negatively regulate PCD (Lee et al., 2017). The leaves of *ANAC080* overexpressing plants display accelerated and rapidly spreading PCD following infection with *Pseudomonas syringae*, while in null mutants cell death spread was delayed. *ANAC080* itself is negatively regulated by a microRNA 164, allowing fine-tuning of the appropriate immune response and ensuring that PCD is tightly controlled (Lee et al., 2017).

Several NAC TFs are also involved in cell death induced by ER stress. The accumulation of misfolded proteins in the ER triggers the unfolded protein response (UPR), a widely conserved pro-survival mechanism (Calfon et al., 2002). However, extreme, or prolonged ER stress can lead to the activation of PCD (Zuppini et al., 2004). Many environmental stimuli, such as salinity, heat, drought, osmotic stress, and pathogens, can evoke the ER stress responses (Park and Park, 2019). In soybean, programmed cell death induced by both ER and osmotic stress was linked to GmNAC30 and GmNAC81 (Faria et al., 2011; Mendes et al., 2013). The GmNAC30 and GmNAC81TFs form homo- or heterodimers and may act as both transcriptional activators or repressors, with their ability to promote PCD linked to transactivation of the *vacuolar processing enzyme* (*VPE*) gene by a NAC81/NAC30 heterodimer (Mendes et al., 2013). VPE is responsible for the caspase-1 activity and may contribute to PCD *via* the activation of vacuolar proteases and subsequent vacuole collapse (Hatsugai et al., 2006). Another NAC, NAC089 was implicated in ER stress induced PCD in *Arabidopsis* (Yang et al., 2014). Similarly to NAC81/NAC30 dimer, ANAC089 promotes the induction of caspase-like activity during ER stress induced PCD, and also appears to regulate other downstream PCD-associated genes including *BAG6* (*Bcl-2-associated athanogene family member*) and *MC5* (*metacaspase 5*). The transcription of *NAC089* is itself promoted by two membrane bound TFs, bZIP28 and bZIP60, highlighting the multiple levels of regulation involved in initiating the PCD cascade (Yang et al., 2014). In rapeseed, four NAC TFs (BnaNAC55, BnaNAC56, BnaNAC87, and BnaNAC103) have separately been shown to be involved in PCD following treatment with diverse abiotic stressors (Niu et al., 2014; Niu et al., 2016; Chen et al., 2017; Yan et al., 2017). In all cases, expression of the respective TF resulted in the development of HR-like lesions, reactive oxygen species (ROS) accumulation, and DNA degradation, however, the molecular mechanisms by which these TFs induce cell death has not been examined.

PCD is commonly induced following severe genotoxic stress in order to protect the organism from deleterious DNA mutations. This process initially involves cell cycle arrest and attempts at DNA repair, with apoptosis initiated if the damage is too severe (Norbury and Zhivotovsky, 2004). In animals this DNA damage response is largely coordinated by p53, a constitutively expressed TF that is stabilized *via* phosphorylation by four DNA damage sensing kinases; ATM, ATR, CHK1, and CHK2 (Lavin and Gueven, 2006). p53 not only induces apoptosis by regulating the transcription of apoptotic genes but also translocates to the mitochondria where it can modulate mitochondrial outer membrane permeabilization (MOMP) *via* direct interactions with pro- and anti-apoptotic proteins (Vaseva and Moll, 2009). Although several key DNA damage response genes such as *ATM* and *ATR* are conserved across plants and animals, *p53* is not (Culligan et al., 2006). Instead, plants have developed a functional homolog of p53, SOG1/ANAC008, which fulfills similar functions in coordinating the DNA damage response (Yoshiyama et al., 2009; Yoshiyama et al., 2014). The root meristematic stem cell niche and its early descendants are hypersensitive to genotoxic stress (Fulcher and Sablowski, 2009), and undergo a selective type of PCD that is mediated by SOG1 and requires *de novo* protein synthesis (Furukawa et al., 2010). More recently it has been established that SOG1/ANAC008 is necessary not only to trigger PCD in these cell populations but also to mediate a regenerative response in meristematic tissue for the stem cell niche replenishment (Johnson et al., 2018). The SOG1 direct targets include genes implicated in response to abiotic stresses and pathogen infection (Ogita et al., 2018). Two of SOG1 targets, *ANAC044* and *ANAC085*, are its closest relatives in the NAC TF family and were suggested to also participate in SOG1-mediated induction of stem cell death (Takahashi et al., 2019). However, it is not clear which key downstream PCD effectors are controlled by SOG1/ANAC008 signaling. Chilling stress was shown to induce DNA damage dependent cell death of columella stem cell daughters (Hong et al., 2017). This highly localized cell death appeared to protect the stem cell niche from chilling stress and improve the root’s ability to withstand the accompanying environmental stresses and resume growth (Hong et al., 2017). Considering the role of SOG1/ANAC008, ANAC044, and ANAC085 in regulation of PCD induced by DNA damage, it would be interesting to test the effect of these TFs on adaptation and survival of roots under the chilling stress.

The role of NAC TFs in lysigenous aerenchyma formation is also slowly emerging. The meta-analysis of quantitative trait loci (QTL) associated with abiotic stress tolerance identified a NAC domain TF as a key candidate gene for aerenchyma formation in barley (*Hordeum vulgare*) under waterlogging conditions (Zhang et al., 2017b). Several NAC TFs were linked to aerenchyma formation also in rice (*Oryza sativa*). For example, transgene overexpression of stress‐inducible *OsNAC5* and *OsNAC9* resulted in enhanced aerenchyma formation in rice, especially under the root-specific promoter, and correlated with enhanced drought and salinity tolerance (Redillas et al., 2012; Jeong et al., 2013). Rice offers an interesting model for further studies delineating the transcriptional regulation of developmental and environmentally induced lysigenous aerenchyma, as this tissue forms constitutively in rice roots but is further induced by flooding (Yamauchi et al., 2013).

## Ethylene Responsive Element Binding Factors Transcription Factors in the Regulation of Plant Programmed Cell Death

The ethylene responsive element binding factors (ERFs) belong to the AP2/ERF superfamily, characterized by the presence of one (in ERF) or two (in AP2) 60-70 residue AP2/ERF DNA binding domains (Nakano et al., 2006). This expansive group of transcriptional regulators display a wide range of roles in responding to various forms of abiotic stress (Mizoi et al., 2012; Li et al., 2017; Najafi et al., 2018). MACD1 and ERF102 are two ERFs linked to phytotoxin induced cell death (Mase et al., 2013) and both act downstream of ethylene signaling and are positive regulators of programmed cell death induced by the phytotoxins AAL and fumonisin B1. ERF TFs are also involved in regulation of HR PCD, for example NbCD1 is an ERF that is expressed in response to multiple HR elicitors, and its conditional expression is sufficient to induce cell death (Nasir et al., 2005). Expression of *NbCD1* also results in high levels of H_2_O_2_ generation, ion leakage, and DNA fragmentation. Additionally, NbCD1 modulates transcription *via* its ERF-associated amphiphilic repression (EAR) motif. NbCD1 positively regulates HR cell death by suppressing the transcription of almost 60 genes, including *HSR203*, a negative regulator of the HR (Nasir et al., 2005). The tobacco transcriptional repressor NtERF3 is another EAR motif containing TF that has been identified as an inducer of HR-associated PCD following Tobacco mosaic virus infection (Ogata et al., 2012). As with *NbCD1*, overexpression of *NtERF3* was sufficient to induce HR-like lesions on tobacco leaves, while deletion of the EAR motif from this TF prevented the HR cell death. Subsequent analysis of the *Arabidopsis*, rice, and tobacco genomes enabled the identification of dozens of closely related group VIII ERF genes (Ogata et al., 2013). Interestingly, overexpression of several group VIII-a ERFs (containing an EAR-motif) induced cell death, while overexpression of group VIII-b ERFs (lacking an EAR-motif) failed to induce cell death morphology in *Arabidopsis* (Ogata et al., 2013). However, the degree of cell death induced by different EAR-motif containing ERFs varied significantly, and the expression of fusion proteins consisting of group VIII-b ERFs fused to EAR motifs also failed to induce cell death, suggesting that the presence of an EAR motif alone is not sufficient to induce a transcriptional program resulting in PCD (Ogata et al., 2013).

ERF TFs are also involved in the regulation of PCD induced by abiotic stress. For example, ERF109 is implicated in salt stress tolerance, acting as a negative regulator of PCD in *Arabidopsis* (Bahieldin et al., 2016). This TF prevents PCD by inducing expression of *Bax-inhibitor 1*, which inhibits the pro-apoptotic Bax protein (Bahieldin et al., 2016). Ethylene is involved in lysigenous aerenchyma formation (Yamauchi et al., 2013) and treatment with ethylene inhibitors decreases aerenchyma formation under hypoxia (Drew et al., 1981; Gunawardena et al., 2001a). ERFs have been linked to both aerenchyma formation and waterlogging tolerance in several species and recently, the PhERF2 TF was found to modulate PCD during waterlogging response in petunia (Yin et al., 2019). Overexpression of *PhERF2* increased survival of waterlogged seedlings while the silencing lines exhibited compromised waterlogging tolerance with increased leaf chlorosis and necrosis. The root cells of *PhERF2* overexpressor plants displayed condensed, moon-shaped nuclei, characteristic of PCD, suggesting that this TF may positively regulate aerenchyma formation (Yin et al., 2019). Multiple transcriptome profiling analyses reported differential expression of ERFs in response to conditions inducing aerenchyma, such as waterlogging and hypoxia (Rajhi et al., 2011; Safavi-Rizi et al., 2020) or in tissues undergoing developmental aerenchyma formation (Yoo et al., 2015; Du et al., 2018). However, functional validation studies are required to determine if the identified ERFs indeed contribute to aerenchyma induction. RAV1 seems to be a promising candidate, as the *RAV1-like* gene was induced specifically in maize cortical cells (aerenchyma‐forming tissue) in response to waterlogging and this up-regulation was blocked upon pretreatment with ethylene perception inhibitor 1-methylcyclo-propene (1-MCP) (Rajhi et al., 2011). *RAV1* was later proposed to underlie Subtol6, a major QTL associated with submergence tolerance in maize (Campbell et al., 2015). The RAV1 TF was also suggested to regulate the initial steps of constitutive aerenchyma formation in sugarcane that involve cell wall polysaccharide modifications (Tavares et al., 2019). This is in line with work by Gunawardena et al. (2001b) who proposed that one of the earliest, ethylene-promoted, changes associated with aerenchyma formation are the alterations to cell wall polysaccharides. The role of RAV1 in regulation of PCD is plausible, as RAV1 overexpression in *Arabidopsis* results in accelerated senescence (Woo et al., 2010) and a *RAV1* homologue was strongly induced in pepper leaves during the early response to pathogen infection, abiotic elicitors, and environmental stresses (Sohn et al., 2006). *RAV1* itself might be regulated post-transcriptionally by microRNAs (Tavares Queiroz de Pinho et al., 2020), allowing tightly controlled expression of its target genes.

## WRKY Transcription Factors in Programmed Cell Death Regulation

WRKY transcription factors are a diverse group of transcriptional regulators that integrate plant responses to environmental stress and regulate development (Bakshi and Oelmuller, 2014). WRKY TFs are categorized by the presence of 60 conserved amino acid residues at the N-terminus (Bakshi and Oelmüller, 2014; Phukan et al., 2016). The WRKY TF family targets genes containing a CRE containing W-box element (TGAC) (Eulgem et al., 2000). Several WRKY TFs are involved in the regulation of cell death during biotic stress. In tobacco, WRKY1 was first identified as a positive regulator of HR PCD, following its phosphorylation and activation by the salicylic acid (SA) induced kinase SIPK (Menke et al., 2005). WRKY18, WRKY40, and WRKY60 also modulate transcription of pathogen responsive genes *via* the formation of homo- or heterodimers (Xu et al., 2006). A triple knockout *Arabidopsis* line lacking all three TFs was more susceptible to infection by *Botrytis cinerea*, a necrotrophic fungal pathogen that promotes host cell death in a HR-like manner. The same KO line displayed increased resistance to *P. syringae*, a bacterial pathogen that is biotrophic during the early stages of infection (Xu et al., 2006). This suggests that this network of WRKY TFs may function to suppress HR cell death during the initial infection, although the transcriptional program they promote to achieve this has not yet been identified. The WRKY52 TF from the grapevine (*Vitis quinquangularis*) has the opposite role, as transgenic expression of *VqWRKY52* in *Arabidopsis* results in significantly greater cell death following infection by both *B. cinerea* and *P. syringae*, and thus increased and reduced susceptibility to the necrotrophic and biotrophic pathogens respectively (Wang et al., 2017). Finally, transient expression of phospho-mimicking mutants of *WRKY7*, *8*, *9*, *11*, *12*, and *14* is sufficient to induce cell death in *Nicotiana benthamiana*, with these TFs appearing to act downstream of a MAPK phosphorylation cascade (Adachi et al., 2015). Interestingly, the degree of cell death induced by these TFs was correlated to their ability to induce a respiratory burst oxidase homologue (RBOH) derived ROS burst, which has previously been shown to be required for resistance to biotic and abiotic stress, and for certain forms of PCD (Xie et al., 2014; Li et al., 2015). However, the relevance of such experiments involving phospho-mutants to physiological HR mechanisms is not clear.

Transcriptomic analyses suggested that WRKY TFs can regulate constitutive and environmentally induced lysigenous aerenchyma induction in rice (Yoo et al., 2015; Viana et al., 2018). However, *WRKY53* and *WRKY33* showed higher expression under submergence conditions in the waterlogging sensitive maize genotypes compared to tolerant lines (Campbell et al., 2015). Further research is therefore required to delineate the role of WRKYs in aerenchyma formation, which may differ between developmentally or environmentally induced aerenchyma. Interestingly, *HaWRKY76*, a divergent transcription factor from sunflower, conferred submergence tolerance when overexpressed in *Arabidopsis*, which in part can be linked to enhanced formation of lysigenous stem aerenchyma (Raineri et al., 2015).

## Other Transcription Factors Contributing to Modulation of Environmentally Induced Programmed Cell Death

Several other TF classes are also likely to contribute to the transcriptional regulation of life-and-death decisions in response to environmental stress. Auxin response factors (ARFs), which bind to auxin response elements (Li et al., 2016) and similarly to other TF families, possess an N-terminal DNA binding domain combined with a C-terminal domain suited to protein-protein interactions (Ulmasov et al., 1997). Although ARFs are typically associated with growth and developmental processes, their involvement in PCD regulation is possible, as supplementation of auxin or auxin analogues has been shown to block PCD following biotic and abiotic stresses such as exposure to the bacterial effector thaxtomin A or photorespiratory induced oxidative stress (Kerchev et al., 2015; Awwad et al., 2019). The molecular mechanisms responsible for this death-suppressing effect and potential involvement of ARFs require further research. The interplay between auxin and ethylene was suggested to regulate aerenchyma formation in maize under waterlogging stress where the auxin associated genes such as *IAA3*, *IAA14*, and *IAA16* were shown to be upregulated in the tolerant genotypes (Thirunavukkarasu et al., 2013). The IAAs are the short-lived, early auxin response proteins that interact with ARFs and inhibit the transcription of their target genes (Luo et al., 2018). The IAAs- and ARFs- dependent auxin signaling was also linked to formation of constitutive aerenchyma in rice (Yoo et al., 2015; Yamauchi et al., 2019).

Another family of TFs linked to plant PCD modulation are the MYBs, a diverse family of eukaryotic transcription modulators with roles in both development and stress responses (Dubos et al., 2010). In *Arabidopsis*, AtMYB30 is a positive regulator of HR cell death, that was initially discovered due to its strong upregulation immediately following infection with HR inducing bacterial effectors (Daniel et al., 1999; Vailleau et al., 2002). The expression of *AtMYB30* is dependent on SA accumulation, and plants with knock-down, knock-out, or overexpression mediated perturbations in AtMYB30 levels in turn display altered SA levels, suggesting that the TF functions at least partially as an SA signaling amplification loop (Raffaele et al., 2006). It has been subsequently shown that AtMYB30 enhances the expression of several genes involved in very long chain fatty acid (VLCFA) synthesis and may also promote PCD by utilizing VLCFAs or their derivatives as cell death messaging molecules (Raffaele et al., 2008). The ectopic expression of rapeseed (*Brassica napus*) *BnaMYB78* in *N. benthamiana* has also been shown to induce a form of HR-like cell death associated with H_2_O_2_ production, although the function of this TF in *B. napus* or indeed of its *Arabidopsis* homologue remain to be investigated (Chen et al., 2016). Many MYB TFs have been proposed as putative regulators of aerenchyma formation by transcriptome profiling studies (Thirunavukkarasu et al., 2013; Valliyodan et al., 2014) and a meta-analysis of major QTL for waterlogging tolerance (Zhang et al., 2017b). During hypoxic treatment of wheat roots, expression of the *TaMyb1*, when analyzed using *in situ* hybridization, was elevated in root epidermal, endodermal, and cortex tissue peripheral to aerenchyma containing cortex (Lee et al., 2006). Further examination of the expression pattern of this TF sequentially during aerenchyma formation may provide more insights into its role in hypoxia responses. The MYB transcription factors *S4877491* and *S4910460* showed higher expression during flooding in waterlogging tolerant soybean genotype with enhanced aerenchyma formation (Valliyodan et al., 2014). Moreover, four MYBs were differentially expressed in rice root tissue forming constitutive aerenchyma (Yoo et al., 2015). However, functional studies are required in order to determine if MYB TFs indeed play a role in the regulation of cell death during aerenchyma formation in response to environmental stimuli.

## Mitochondria, Transcription Factors, and Cell Fate Regulation

The role of mitochondria in plant PCD has been widely documented (Van Aken and Van Breusegem, 2015) although details of this involvement have not yet been fully elucidated. Mitochondria act as stress sensing organelles, with both extrinsic (environmental) and intrinsic (cellular) stimuli affecting the mitochondrial respiratory status (Schwarzlander and Finkemeier, 2013). Such changes can trigger signaling pathways, that either regulate mitochondria directly, which may result in events leading to PCD activation (Garmier et al., 2007; Gao et al., 2008; Scott and Logan, 2008; Bi et al., 2009; Wu et al., 2015; Zancani et al., 2015), or induce changes to nuclear gene expression *via* retrograde signaling (Rhoads, 2011; Schwarzlander and Finkemeier, 2013). The output of mitochondrial retrograde signaling not only feeds back to the mitochondrion but also regulates the functions of other cellular compartments (Schwarzlander et al., 2012; Schwarzlander and Finkemeier, 2013), thereby ensuring a coordinated response to environmental or intrinsic perturbations. The role of mitochondrial retrograde signaling in fine-tune regulation of cell fate decisions in plants is emerging, with transcription factors mediating some of the key pathways. Stress responsive mitochondrial proteins were identified by transcriptomic meta-analyses of the mitochondrial protein transcript abundance under a variety of stress conditions or during genetically or chemically induced mitochondrial dysfunction (Van Aken et al., 2009b; Schwarzlander et al., 2012; Wang et al., 2018). Alternative oxidase (AOX), probably the most widely studied stress induced mitochondrial protein and a classical marker of mitochondrial retrograde signaling (Van Aken et al., 2009a; Wang et al., 2018), has been implicated in the negative regulation of PCD response. AOX is a non-proton-pumping, terminal oxidase in the mitochondrial electron transport chain (ETC) (Vanlerberghe, 2013). By uncoupling the electron flow and ATP production, AOX acts as a safety valve, preventing over-reduction of ETC components and dampening the generation of O_2_^−^ and nitric oxide in the mitochondria (Vanlerberghe, 2013). Unsurprisingly for a regulator of mitochondrial and cellular homeostasis, numerous studies report stress-induced PCD phenotypes in plants with altered AOX levels in response to miscellaneous abiotic and biotic factors (Ordog et al., 2002; Lei et al., 2003; Mizuno et al., 2005; Amirsadeghi et al., 2006; Kiba et al., 2008; Li and Xing, 2011; Liu et al., 2014). The pro-survival role of AOX conserved across the plant kingdom; it was recently shown to protect the unicellular algae *Chlamydomonas reinhardtii* from cell death induced by high light (Kaye et al., 2019) and *AOX* isoforms are induced by chemical and environmental stresses in cereal species such as rice and barley (Wanniarachchi et al., 2018). Indeed, the modulation of the AOX pathway has been recently proposed to offer crop protection against the challenges imposed by climate change (Florez-Sarasa et al., 2020). More recently, another stress-responsive mitochondrial protein has been linked to PCD regulation. *Om66* (*outer mitochondrial membrane protein of 66 kDa*), previously annotated as *AtBCS1* (*cytochrome BC1 synthase 1*), is induced by SA (Ho et al., 2008), mitochondrial and chloroplast perturbations (Van Aken and Whelan, 2012) and by biotic stress signals and UV light (Zhang et al., 2014). Interestingly, the OM66 transcript is also rapidly induced by the touch stimulus (Van Aken et al., 2016a), a mechanism that has not yet been investigated in the PCD context. *Arabidopsis thaliana* protoplasts treated with UV light exhibited increased cell death rates when *OM66* was overexpressed, and reduced cell death in the loss of function mutants; the *OM66* overexpressor (*OM66* OE) plants also demonstrated accelerated senescence and increased drought tolerance (Zhang et al., 2014). The *OM66* OE was more tolerant to the biotrophic *P. syringae* but showed increased susceptibility to the necrotroph *B. cinerea* (Zhang et al., 2014). In line with the observed PCD phenotypes, the gene expression analysis revealed changes in pathogen defense signaling, cell death, and senescence in *OM66* OE lines (Zhang et al., 2014).

While the molecular mechanisms behind the regulation of cell death-suppressing AOX and cell death-promoting OM66 are still being uncovered, several TFs were demonstrated to play a role. There is an overlap between *AOX* and *OM66* regulation in response to mitochondrial dysfunction, although the rapid touch induction of *OM66* seems to be mediated by a distinct signal transduction pathway (Van Aken et al., 2016a). Under non-stress conditions, the TF abscisic acid insensitive 4 (ABI4) acts as *AOX1a* repressor in *A. thaliana*, with de-repression induced by rotenone or abscisic acid (ABA) itself (Giraud et al., 2009) suggesting that additional ABA response factors may regulate *AOX1a*, both positively and negatively (Wang et al., 2018). MYB29 is a general negative regulator of mitochondrial stress response, repressing both *AOX1a* and *OM66* indirectly *via* regulation of the expression of various *ERF* and *WRKY* transcription factors (Zhang et al., 2017a). The expression of *OM66* and *AOX1a* under mitochondrial stress conditions is also regulated by WRKY transcription factors, with likely functional redundancy suggested between them (Van Aken et al., 2013; Van Aken et al., 2016a). Knockout and overexpressor studies suggest that under stress conditions such as high light or actinomycin treatment, WRKY40 generally acts as a repressor of genes commonly affected by both chloroplast and mitochondrial perturbation, while WRKY63 is their activator (Van Aken et al., 2013). Interestingly, under no stress conditions, *OM66* but not *AOX1a* was induced in *WRKY63* OE line, highlighting differences in the pathways involved in regulation of these mitochondrial stress signaling genes (Van Aken et al., 2013). ANAC017 is an ER-tethered transcription factor and among the best characterized positive regulators of mitochondrial retrograde signaling (Ng et al., 2013). Once released from the ER, ANAC017 modulates the transcription of hundreds of nuclear and mitochondrial encoded genes, involved in energy metabolism, redox balance, mitochondrial fission, and hormone signaling, with both *AOX1a* and *OM66* among its target genes (Ng et al., 2013; Van Aken et al., 2016a). ANAC017 creates a positive feedback loop by inducing the expression of another ER bound TF, ANAC013, which activates its own expression, as well as promoting expression of the same target genes as ANAC017 (Van Aken and Pogson, 2017). The *anac017* knockout plants show a complete loss of *OM66* and *AOX1a* induction by mitochondrial perturbation, while the rapid touch induction of OM66 remains unchanged in *anac017* background, and instead is regulated by a complex signaling network involving WRKY40 and WRKY15, which themselves are also induced by touch, suggesting a negative feedback loop (Van Aken et al., 2016a; Xu et al., 2019). Moreover, the presence of OM66 is required for the touch induction of *WRKY40* (Xu et al., 2019). While the PCD rates induced by environmental factors have not been investigated in *ANAC017* mutant/transgenic lines, the overexpression of ANAC017 causes reduced cell viability and expansion, as well as early senescence, likely due to disturbed mitochondrial signaling (Meng et al., 2019). Moreover, the *anac017* knockout mutants are more sensitive to drought stress (Ng et al., 2013) and submergence (Meng et al., 2020) and show increased accumulation of ROS under stress conditions (Meng et al., 2020). Additionally, the double mutants with loss of function in both *ANAC017* and mitochondrial RNA polymerase (resulting in reduced activity of ETC complexes I and IV) display distinctive PCD-associated lesions (Van Aken et al., 2016b).

To conclude, mitochondria integrate stress signals and environmental stimuli resulting in perturbation of mitochondrial function (Rhoads, 2011; Schwarzlander and Finkemeier, 2013). The mitochondrial stress responsive proteins, such as AOX1a and OM66, can modulate cell fate decisions, and are regulated by complex, partially overlapping retrograde signaling networks involving numerous TFs, including WRKY15, WRKY40, MYB29 and ABI4, WRKY63, ANAC013, ANAC017. Detailed PCD phenotyping, in both abiotic and biotic context, is required for plants with reduced/enhanced expression of these TFs in order to further elucidate their role in modulation of cell death pathways, ideally in combination with monitoring of mitochondrial retrograde signaling. Methods such as root hair assay (Kacprzyk et al., 2014; Kacprzyk et al., 2016) or measurements of aerenchyma formation may provide useful tools to easily obtain quantitative information on the rates of PCD induced by numerous environmental stimuli in such mutants/transgenes. Finally, it remains to be established if the touch signaling, involving rapid upregulation of cell death promoting *OM66*, and activation regulatory network that mediates the responses to abiotic and biotic stresses, has an effect on plant’s susceptibility to subsequent PCD triggers by environmental stimuli.

## Conclusion and Perspectives

Our understanding of PCD regulation in response to environmental stimuli is expanding. Increasing numbers of TFs are implicated in the transcriptional control of stress-induced cell fate decisions in plants. Details of the signaling pathways associated with the individual TFs are also emerging (**Table S1**), however, an integrative (meta)-analysis of gene regulatory network activated during PCD induced by abiotic and biotic stresses is required. Approaches allowing quantitative assessment of rates and timing of PCD, occurring in response to abiotic and biotics stresses will support further elucidation of TF mediated control of cell death processes in plants. The complex regulatory networks activated in response to environmental stresses need to be studied in the PCD context, including delineation of the cooperative action between individual TFs and detailed characterization of their targetomes. Furthermore, exploring the interplay between microRNAs and TFs implicated in stress induced PCD will reveal another layer of gene regulatory network(s) involved. Such research will be expedited by technological advances, like ultra-affordable transcriptomics (Alpern et al., 2019) and resources such as AtTORF-Ex seed collections (*Arabidopsis thaliana* *T*F *ORF* over-*Ex*pression) (Weiste et al., 2007). Cautious, fine-tuned control of PCD activation is required in plants to successfully cope with the environmental challenges they cannot escape. In particular, recent advances in the understanding of organellar retrograde signaling highlight the ability of TFs to act as molecular switches between pro-death and pro-survival responses. Further research into these PCD regulatory nodes is thus crucially important for future crop improvement strategies.

## Author Contributions

JK and RB conceived an original idea for a review. RB, JS, OS, and JK drafted the initial version and RB prepared the figure. All authors contributed to the article and approved the submitted version.

## Funding

RB was funded by University College Dublin PhD Advance grant awarded to JK and PM. JS was funded by the School of Biology and Environmental Science. OS was funded by Environmental Protection Agency-Irish Research Council (EPA-IRC) Postgraduate Scholarship. YJ was funded by Newman Fellowship Programme and Council for At-Risk Academics (CARA).

## Conflict of Interest

The authors declare that the research was conducted in the absence of any commercial or financial relationships that could be construed as a potential conflict of interest.
