# Supplementary material for: Stressed to Death: The Role of Transcription Factors in Plant Programmed Cell Death Induced by Abiotic and Biotic Stimuli
Source: Front Plant Sci. 2020 Aug 12;11:1235. doi: 10.3389/fpls.2020.01235 (PMC7434935; doi:10.3389/fpls.2020.01235)
Supplement: Supplementary file 1 [file Table_1.docx]

**Table S1. Known elements of signaling networks associated with TFs with experimentally validated role in stress-induced PCD.** TFs promoting PCD are highlighted in red, TFs supressing PCD are highlighted in blue. Only TFs with experimentally validated role in PCD regulation are presented. The asterisks (*) indicate direct target genes, OE – overexpressor.

| **Family** | **TF** | **Stress Type** | **Upstream regulators relevant to PCD** | **Interacting/Targeted (*) TFs in PCD context** | **Regulated/Targeted (*) PCD related genes** | **Consensus target sequence** | **Resources and datasets** |
| --- | --- | --- | --- | --- | --- | --- | --- |
| NAC | GmNAC81 | ER Stress  (Faria et al., 2011; Mendes et al., 2013) |  | GmNAC30  (Mendes et al., 2013) | *VPE*-1*  (Mendes et al., 2013) | TGTGTT  (Mendes et al., 2013) | ChIP Assay  (Mendes et al., 2013) |
|  | GmNAC30 | ER Stress (Mendes et al., 2013) |  | GmNAC81  (Mendes et al., 2013) | *VPE*-1*  (Mendes et al., 2013) | TGTGTT  (Mendes et al., 2013) | ChIP Assay  (Mendes et al., 2013) |
|  | AtNAC089 | ER Stress  (Yang et al., 2014) | bZIP28, bZIP60  (Yang et al., 2014) | NAC094*  (Yang et al., 2014) | *MC5**, *BAG6**  (Yang et al., 2014) |  | ChiP Assay  Microarray (*NAC089D-MYC* expressing plants)  (Yang et al., 2014) |
|  | AtNAC008/ SOG1 | Genotoxic Damage (Yoshiyama et al., 2009; Yoshiyama et al., 2014) | ATM, ATR (Yoshiyama et al., 2013) | NAC103, ANAC044*, ANAC085*  (Ryu et al., 2018; Ogita et al., 2018; Takahashi et al., 2019) | *PLA2A**  (Bourbousse et al., 2018) | CTT (N)_7_AAG  (Ogita et al., 2018) | Microarray (*sog1-1* plants post genotoxic treatment);  ChIP Assay  (Ogita et al., 2018) |
|  | AtNAC017 | Mitochondrial Stress  (Ng et al., 2013; Van Aken and Pogson, 2017) | Unknown rhomboid protease  (Ng et al., 2013) | ANAC013*, ATAF1, ANAC019, ANAC032  (Ng et al., 2013) | *AOX1a**, *OM66*  (Ng et al., 2013; De Clercq et al. 2013);  Genes with a cell death related GO term annotation are upregulated in *ANAC017* OE plants  (Meng et al., 2019) | CA(C/A)G  (Ng et al., 2013) | RNA-seq (*ANAC017* OE and *anac017* KO lines)  (Meng et al., 2019);  RNA-seq (*rao2-1* and *anac017-2* plants post submergence and desubmergence)  (Meng et al., 2020) |
|  | OsNAC4 | Biotic Stress (Kaneda et al, 2009) |  |  | *OsHSP90*, *IREN*  (Kaneda et al., 2009) |  | Microarray (*OsNAC4*-RNAi; control and post *Acidovorax avenae* N1141 inoculation)  (Kaneda et al., 2009) |
|  | AtNAC080 | Biotic Stress  (Lee et al., 2017) | *miR164*  (Lee et al., 2017) | WKRY40*, WRKY54*  (Lee et al., 2017) | *LURP1**  (Lee et al., 2017) | ACAAGCAAC  (Lee et al., 2017) | Microarray (*nac4-1* KO and *35S:NAC4* OE plants)  (Lee et al., 2017) |
| WRKY | NbWRKY1 | Biotic Stress  (Menke et al., 2005) | SIPK  (Menke et al., 2005) |  |  | TGAC  (Yang et al., 1999; Menke et al., 2005) |  |
|  | VqWRKY52 | Biotic Stress (Wang et al., 2017) |  |  |  |  |  |
| MYB | AtMYB30 | Biotic Stress  (Daniel et al., 1999; Vailleau et al., 2002) | AtsPLA2ɑ, MIEL1, SBT5.2  (Froidure et al., 2010; Marino et al., 2013; Serrano et al., 2016) |  | Acyl CoA elongase complex  (Raffaele et al., 2008) | GTTTGTT / GTTGTTGT  (Li et al., 2009; Mabuchi et al., 2018) | Microarray (*MYB30* OE and *MYB30 anti-sense* plants; control and post *Xcc*147 inoculation)  (Raffaele et al., 2008);  RNA-Seq (*myb30-2* and *MYB30-*OE plants; control and H_2_O_2_ treatment)  (Mabuchi et al., 2018) |
| ERF | NbCD1 | Biotic Stress (Nasir et al., 2005) |  |  | *HSR203*, *PR*-*1a*, *PR*-*1b*  (Nasir et al., 2005) |  | SuperSAGE (*NbCD1-HA* OE)  (Nasir et al., 2005) |
|  | NtERF3 | Biotic Stress (Ogata et al., 2012) | NtSIPK-NtWRKY1  (Ogata et al., 2015) |  |  | GCC Box: TAAGAGCCGCC  (Ohme-Takagi and Shinshi, 1995) |  |
|  | NuMACD1 | Biotic Stress (Mase et al., 2013) |  |  |  | GCC Box: AGCCGCC  (Mase et al., 2013) |  |
|  | AtERF102 | Biotic Stress  (Mase et al., 2013) |  |  |  | GCC Box: AGCCGCC  (Mase et al., 2013) | Microarray (*ERF102* OE)  (Mase et al., 2013) |
|  | NbERF109 | Salinity  (Bahieldin et al., 2016) |  |  | *Bax Inhibitor1*  (Bahieldin et al., 2016) | GCC Box: GCCGCC  (Cai et al., 2014) |  |
|  | PhERF2 | Waterlogging  (Yin et al., 2019) |  |  |  | ATCTA  (Yin et al., 2019) |  |
